# Drug concentrations in the serum and cerebrospinal fluid of patients treated with norvancomycin after craniotomy

**DOI:** 10.1007/s10096-016-2803-9

**Published:** 2016-10-13

**Authors:** Y. Wu, J. Kang, Q. Wang

**Affiliations:** 1 Respiratory and Critical Care Medicine, Beijing Anzhen Hospital, Capital Medical University, Beijing, 100029 People’s Republic of China; 2 Department of Neurosurgery, Affiliated Cancer Hospital of Zhengzhou University, Zhengzhou, 450008 People’s Republic of China; 3 Intensive Care Unit, Beijing Tiantan Hospital, Capital Medical University, Beijing, 100050 People’s Republic of China

## Abstract

Intracranial infection by gram-positive cocci is commonly found after craniotomy. Norvancomycin was independently developed in China, and had demonstrated therapeutic capability against gram-positive infection. This study investigated the serum and cerebrospinal fluid (CSF) concentrations in patients that received intravenous injection of norvancomycin after craniotomy. Patients with an indwelling catheter in the operational area/ventricle after craniotomy were administered norvancomycin by two approaches: (1) The conventional group consisted of 14 cases that were infused with 0.8 g norvancomycin for 1 h, every 12 h; (2) The continuous administration group consisted of 14 cases that were infused with 0.8 g norvancomycin for 1 h, and then another 0.4 g for 11 h with extended infusion, followed by continuous infusion of 0.4 g norvancomycin for 12 h. Samples of serum and CSF were collected at different time-points to measure norvancomycin levels after administration. In the conventional and continuous administration groups, the peak serum concentrations of norvancomycin were 55.52 ± 26.04 and 59.22 ± 41.88 mg/L, respectively, while those at 24 h were 8.21 ± 6.04 and 8.01 ± 4.17 mg/L, respectively. Meanwhile, peak CSF concentrations were 16.31 ± 11.15 and 8.82 ± 8.91 mg/L, respectively, while those at 24 h were 6.12 ± 2.34 and 6.24 ± 4.38 mg/L, respectively. This preliminary study showed that for the early administration of standard doses of norvancomycin post-neurosurgery, the CSF concentration in both the conventional and continuous administration groups reached or exceeded the 90 % minimum inhibitory concentration (MIC_90_, 2 mg/L) of target bacteria such as methicillin-resistant *Staphylococcus aureus* (MRSA).

## Background

Intracranial infection after craniotomy, one of the most severe postoperative complications, is a challenge in neurosurgical treatment. Its high incidence impacts outcomes of patients dramatically [1, 2]. The leading pathogens of surgical site infections in postneurosurgical patients are Gram-positive bacteria, especially S. aureus. Epidemiological studies of nosocomial infection in our unit revealed that gram-positive bacteria were still common pathogens among intracranial infections after craniotomy, which mainly included methicillin-resistant coagulase negative staphylococci (MRCoNS) and *Staphylococcus aureus* [3, 4].

Vancomycin is a glycopeptides antibiotic that has been widely used with activity primarily directed toward Gram-positive pathogens, such as *Staphylococcus aureus*, methicillin-resistant *Staphylococcus aureus*, *Staphylococcus epidermis*, and *Enterococcus faecalis* [5, 6]. Therefore, Vancomycin currently is commonly used as a first-line empiric antibiotic therapy to prevent and treat these intracranial infections [6, 7], despite its low CSF permeability.

Norvancomycin, a glycopeptide antimicrobial agent, was independently developed by Chinese scientists, with a comparable antibacterial spectrum and antibacterial activities to those of vancomycin [8]. Norvancomycin had been widely used in neurosurgery in China, although pharmacokinetics-related studies conducted with this antimicrobial after craniotomy have been rarely reported. Our studies preliminarily confirmed that damage of the blood–brain barrier (BBB) by neurosurgery was likely to increase the concentrations of vancomycin and other antibiotics in the CSF of post-operative patients [9, 10].

This study was designed to investigate whether there is an increased CSF penetration of norvancomycin into the cerebrospinal fluid increases after BBB is impaired following craniotomy, and whether continuous infusion time affects drug concentration.

### Study design

This was a prospective observational study.

## Materials and methods

### Patients

The study was approved by the Research Ethics Committee in Tiantan Hospital, Capital Medical University (Beijing, China,KY2014-014-02, June 4, 2014). Written informed consent was obtained from all patients or their healthcare surrogates prior to enrollment in the study.

### Inclusion criteria

Criteria for inclusion were neurosurgical patients (18–70 years old, entering the ICU of Beijing Tiantan Hospital) with an indwelling ventricular drainage pipe after neurological surgery who were treated with norvancomycin for prevention of intracranial infection between June and October in 2014.

### Exclusion criteria

Criteria for exclusion were patients that had a history of vancomycin or norvancomycin allergies; patients that had received antibiotics against gram-positive bacteria such as MRSA within 7 days before enrollment; patients that were in an agonal state, deep coma, absence of spontaneous breathing, or that had suffered from severe dysfunction of the heart, lung, liver, kidney or other organs; pregnant women in a perinatal stage; and patients that had to combine drug therapy, which might increase drug toxicity, including aminoglycosides, salicylates and blood-lipid regulation drugs, among others.

### Administration norvancomycin and specimen collection

We maintained continuous observation of 14 patients with conformance to the conventional drug administration, and continuous observation of 14 cases of giving continuous dosing. Norvancomycin solution was formulated using norvancomycin (North China Pharmaceutical Group Corporation, China, 400 mg/bottle) and 0.9 % normal saline. Patients in the conventional group were infused with 0.8 g norvancomycin for 1 h every 12 h. In addition, the continuous administration group consisted of 14 cases that were administrated with 0.8 g norvancomycin for 1 h, with an extended infusion of 0.4 g for 11 h, followed by continuous infusion of 0.4 g for 12 h.

Venous blood and CSF were sampled from both groups at the following time points after administration. For the conventional group, 1–1.5 ml CSF and 2–2.5 ml blood sample specimens were drawn at 1 h following the start of an infusion of norvancomycin (ending) for 2, 3, 4, 6, 12, 13, 14 and 24 h. For the continuous administration group, samples were drawn at 1 h after administration (ending), and at 2, 3, 4, 6, 12 and 24 h, following which, samples were rapidly centrifuged at 3 500 rpm for 3 min, where the supernatant was stored frozen at −20 °C and transferred to the pharmacology laboratory of the hospital as soon as possible, and then stored in a −70 °C freezer and ultimately transferred to the central laboratory of the Capital Medical University for uniform testing.

### Determination of norvancomycin concentration

#### Drugs and agents

We used standard vancomycin (i.e., as obtained from The National Institute for the Control of Pharmaceutical and Biological Products; batch number: 0360-200301, and equivalent in dose to 1,007 U/mg), standard norvancomycin (The National Institute for the Control of Pharmaceutical and Biological Products; batch number: 130338-200303, and equivalent to 914 U/mg, content 83.4 %), acetonitrile (Fisher-Scientific, USA, chromatographic pure grade), and analytical reagents, including potassium dihydrogen phosphate (KH_2_PO_4_), phosphate, perchloric acid and dichloromethane and deionized water (Millipore Direct+Q).

#### Instruments

We used high performance liquid chromatography (HPLC; Waters, USA) using a 1525 high-pressure infusion system, 717 sampling system, equipped with a 2487 ultraviolet detector and a Waters Breeze chromatographic work station. We also used an Eppendorf high-speed centrifuge, a Stuart turbine mixer, and a Millipore Direct+Q pure-water system (Millipore Ltd., United States).

### Experimental methods

#### Chromatographic conditions

The chromatographic column used was a Sapax C18 column, USA (250 × 4.6 mm, 5 μm); the mobile phase was acetonitrile 0.05 mol/L KH2PO4 buffer (pH = 3.3) at a ratio of 10:90, with a measured wavelength of 230 nm, a velocity of 1.0 ml/min, a column temperature of 30 °C, and a sample volume of 10 μl.

#### Preparation of control solution

For this preparation, 0.0164 g standard norvancomycin was accurately weighed and placed into a 25 ml volumetric flask, dissolved in purified water to a final concentration of 657.3 mg/L control solution, which was double diluted to obtain serial stock solutions at concentrations of 328, 164, 80, 40, 20 and 10 μg/ml. Then, 0.0184 g vancomycin was accurately weighed and placed into a 25 ml volumetric flask, and then dissolved in purified water to obtain a 736.0 mg/ml internal stock solution.

#### Management of plasma/cerebrospinal fluid specimens

In these studies, 0.5 ml plasma/cerebrospinal fluid was placed into a test-tube, each of which had 60 μl of distilled water, 30 μl of an internal stock solution, and 170 μl 10 % perchloric acid added. Then specimens were vortexed for 40 s and centrifuged at 4 000 rpm for 10 min, from which the supernatant was collected and 800 μl dichloromethane was added. This was then vortexed for 1 min and centrifuged at 4000 rpm for 10 min, from which the supernatant was collected for sampling.

#### Preparation of standard curves

In these studies, 0.5 ml of plasma/cerebrospinal fluid specimens was accurately drawn into a test tube, each of which was tested against the formulated serial stock solution of norvancomycin to obtain norvancomycin mass concentrations of 131.46, 65.73, 32.87, 16.43, 8.22, 4.11 and 2.05 μg/ml, following which, the above mentioned management was applied to each test-tube, followed by testing according to preset chromatographic conditions. The peak areas of the norvancomycin and internal label were recorded, and then regression analyses were performed using the weighted least squares method by taking the ratio of the peak area of norvancomycin over that of vancomycin as the horizontal (X) axis, and the mass concentration of norvancomycin as the longitudinal (Y) axis, thereby obtaining the regression equation of the plasma as follows:

$$ {\mathrm{Y}}_1=22.896{\mathrm{X}}_1+1.559,\;{\mathrm{r}}_1=0.9994 $$

Wherein the linear range of norvancomycin in the plasma was 2.05–131.46 μg/ml and the lower limit of detection was quantified as 2.05 μg/ml. The regression equation of CSF specimens was as follows:

$$ {\mathrm{Y}}_2=51.192{\mathrm{X}}_2-6.533,\;{\mathrm{r}}_2=0.996 $$

Wherein the linear range of norvancomycin in CSF was 2.05–131.46 μg/ml and the lower limit of detection was quantified as 2.05 μg/ml.

### Statistical analyses

All the data were analyzed by Microsoft Office Excel 2013. The norvancomycin concentrations in the serum and CSF specimens at each time point were calculated, and the data were expressed as mean ± standard deviation ($\overline{x}$ ± SD). The concentration-time curves were drawn by taking the time as the horizontal axis and the norvancomycin concentration as the longitudinal axis. In addition, the ratios of the area under the serum concentration curve over the MIC_90_ of MRSA were calculated, with the intention of comparing the relationships of norvancomycin concentrations in both serum and CSF specimens with the corresponding bacterial MIC_90_ at each time-point, where the CSF permeability of the norvancomycin was described using the ratio of CSF and serum concentration peaks as well as the ratio of the areas under the curve.

## Results

Twenty-eight patients were enrolled in the study, of which the conventional group consisted of ten male and four female study subjects with a mean age of 40 ± 17 years and a mean weight of 73 ± 13 kg. Meanwhile the continuous administration group consisted of 11 males and three females, with a mean age of 47 ± 14 years and a mean weight of 75 ± 10 kg. Drugs that might increase drug toxicity including aminoglycosides, salicylates and blood-lipid regulatory drugs were not given to any of the patients during administration. Basic information and clinical chemistry measurements are listed in Tables 1, 2, and 3.

**Table 1:** Patient characteristics in conventional groups

Case	Diagnosis	Drainage tube position	Antibiotics before administration
1	Central neurocytoma of left ventricle	Ventricles	Intraoperative 2 g ceferiaxone^a^
2	Glioblastomas of right forehead	Operational cavity drainage	Piperacillin sodium and Sulbactam sodium: 5gQ8h
3	Right frontotemporal glioma	Operational cavity drainage	Intraoperative 1.5 g cefuroxime
4	Meningioma of left petrosal apex	Subdural drainage	Intraoperative 2 g ceferiaxone
5	Meningioma of left ventricle	Operational cavity drainage	Intraoperative 1 g ceferiaxone
6	Left cerebral arteriovenous malformation	Hematoma cavity	Ceftazidine: 2gQ8h
7	Bleeding of cerebellar glioma, ventriculopuncture drainage	Ventricles	Intraoperative 2 g ceferiaxone
8	Right frontal clear cell carcinoma	Operational cavity drainage	Intraoperative 2 g cefuroxime
9	Right frontotemporal glioma	Operational cavity drainage	Intraoperative 1.5 g cefuroxime
10	Fracture of left cerebral artery aneurysm,SAH	Ventricles	Ceftazidine: 2gQ8h
11	Left petroclival meningioma	Operational cavity drainage	Ceftazidine: 2gQ8h
12	Petroclival meningioma	Operational cavity drainage	Sulperazone: 1.5gQ6h
13	Central neurocytoma	Operational cavity drainage	
14	Astrocytoma of thalamus and midbrain	Ventricles	Ceftazidine: 2gQ8h

^a^Well as cerebrospinal fluid penetration rate, ceftriaxone, widely used in China

**Table 2:** Patient characteristics in continuous groups

Case	Diagnosis	Drainage tube position	Antibiotics before administration
1	Glioma in corpus callosum	Operational cavity drainage	Intraoperative 3 g cefuroxime
2	Parasellar meningioma	Ventricles	Intraoperative 1 g ceferiaxone
3	Pineal tumor of corpus callosum	Operational cavity drainage	Intraoperative 2 g ceferiaxone
4	Central neurocytoma of right ventricle	Ventricles	Intraoperative 1 g ceferiaxone
5	Craniopharyngioma	Operational cavity drainage	Intraoperative 1.5 g cefuroxime
6	Neurilemmoma of left petrosal apex	Operational cavity drainage	Piperacillin sodium and Sulbactam sodium: 5gQ8h
7	Glioma of left tempus sinistrum	Operational cavity drainage	Ceftazidine: 2gQ8h
8	Neurilemmoma of left CPA	Operational cavity drainage	–
9	Craniopharyngioma	Operational cavity drainage	Intraoperative 1.5 g cefuroxime
10	Right frontal astrocytoma	Subdural drainage	–
11	Glioma of right thalamus	Ventricles	Ceftazidine: 2gQ8h
12	Craniopharynglioma	Operational cavity drainage	–
13	Deutocerebral glioma	Operational cavity drainage	
14	Cerebellar meningioma	Operational cavity drainage	–

**Table 3:** Patient characteristics in two groups

Characteristics	Conventional (*N* = 14)	Continuous (*N* = 14)	*P* value
Age, years	40 ± 17	47 ± 14	0.264
Gender, % male	71.4 (10/14)	78.6 (11/14)	1.000
Weight, kg	72.9 ± 12.6	75.4 ± 9.9	0.564
Creatinine, μg/ml	51.6 ± 12.6	53.8 ± 17.1	0.709
Day after neurosurgery^a^	1.3 ± 0.5	1.5 ± 1.1	0.596

^a^One day represented the same day after neurosurgery

In the conventional group, serum levels of norvancomycin peaked with a value of 55.52 ± 26.04 mg/L at 1 h after infusion, which decreased to 10.62 ± 10.33 and 8.21 ± 6.04 mg/L by 12 and 24 h, respectively. By contrast, levels of norvancomycin in the CSF peaked with a value of 16.31 ± 11.15 mg/L by 3 h after administration, which decreased to 8.32 ± 5.16 and 6.12 ± 2.34 mg/L by 12 and 24 h, respectively.

Meanwhile, in the continuous administration group, serum levels of norvancomycin peaked with a value of 59.22 ± 41.88 mg/L at 1 h after infusion, which decreased to 8.27 ± 5.7 5 and 8.01 ± 4.17 mg/L by 12 and 24 h, respectively. By contrast, the levels in the CSF peaked with a value of 8.82 ± 8.91 mg/L by 4 h after administration, which decreased to 3.28 ± 1.64 and 6.24 ± 4.39 mg/L by 12 and 24 h, respectively.

**Fig. 1:**
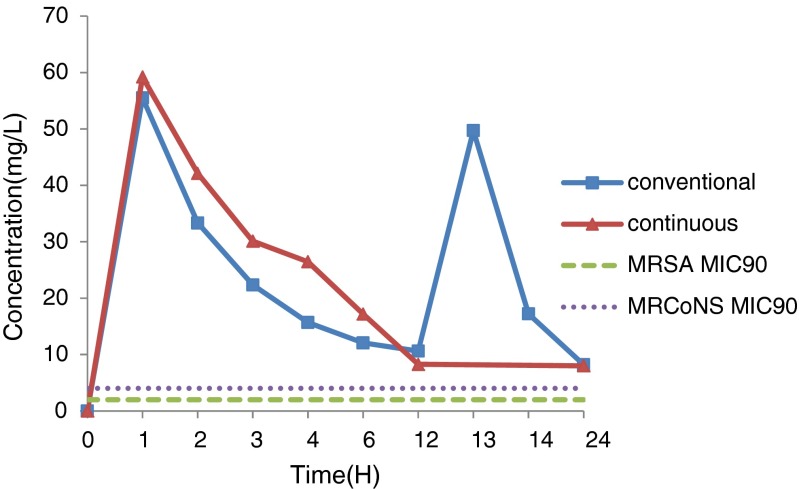
Serum concentration-time curve of norvancomycin in the conventional and continuous group

**Fig. 2:**
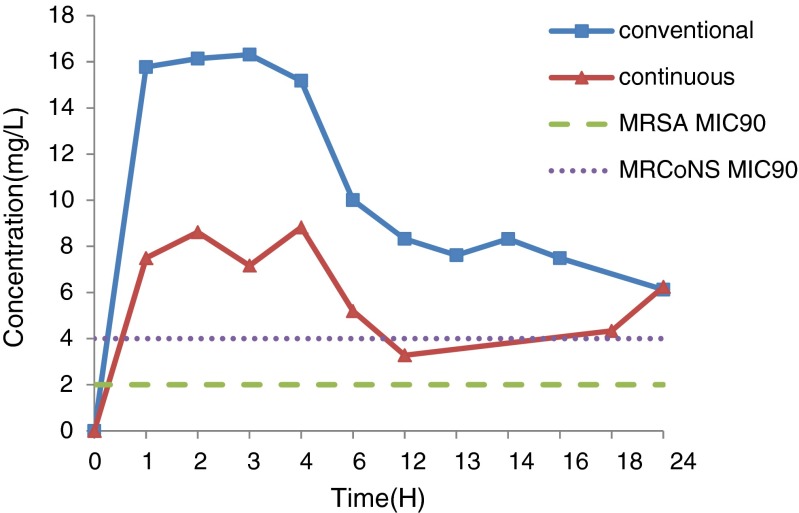
CSF concentration-time curve of norvancomycin in the conventional and continuous group

In the conventional group, penetration of the CSF by the drug that was obtained using the ratios of the peak concentrations during the periods of 1–12 and 12–24 h were 29.4 and 16.7 %, respectively. By contrast, in the continuous administration group, the CSF penetration by the drug that was obtained by using the ratio of the peak concentration and the ratio of the areas under the curves during the 1–12 and 12–24 h were 14.9 and 34.3 %, respectively. In addition, taking the MIC_90_ of the target bacteria like MRSA as 2 mg/L [6], the areas under the serum concentration-time curve AUC_0–24_/MIC_90_ were 193 and 181 in the conventional and continuous administration groups, respectively. The drug concentrations at various time-points are shown in Tables 4 and 5, and Figs. 1 and 2, which illustrate the concentration-time curves of the norvancomycin in both groups.

**Table 4:** Drug concentrations in the serum and CSF of the conventional group

Time (hours)	Norvancomycin (x ± SD)(n)(range)	
	Serum	Cerebrospinal fluid
Prior to administration	0	0
1	55.52 ± 26.04(8) (8.79–91.84)	15.78 ± 8.63(7) (4.03–27.05)
2	33.33 ± 23.52(11) (6.07–67.21)	16.14 ± 9.57(11) (3.03–26.34)
3	22.35 ± 20.37(10) (3.38–59.13)	16.31 ± 11.15(9) (3.48–37.95)
4	15.68 ± 14.77(5) (3.44–39.65)	15.18 ± 16.88(6) (3.37–48.66)
6	12.07 ± 7.50(5) (6.10–24.99)	10.00 ± 7.88(4) (2.80–21.21)
12	10.62 ± 10.33(10) (2.15–36.88)	8.32 ± 5.16(12) (2.68–19.75)
13	49.73 ± 35.31(5) (4.77–102.48)	7.62 ± 3.78(3) (4.49–11.81)
14	17.22 ± 6.52(3) (11.93–24.50)	8.32 ± 6.34(3) (3.19–15.41)
24	8.21 ± 6.04(5) (2.10–17.13)	6.12 ± 2.34(5) (3.15–9.24)

**Table 5:** Drug levels in the serum and CSF of the continuous administration group

Time (hours)	Norvancomycin (x ± SD)(n)(range)	
	Serum	Cerebrospinal fluid
Prior to administration	0	0
1	59.22 ± 41.88(10) (26.96–160.59)	7.49 ± 3.62(9) (2.50–12.37)
2	42.13 ± 30.11(9) (7.80–108.90)	8.62 ± 3.71(9) (3.41–13.56)
3	30.11 ± 18.75(10) (7.08–72.41)	7.17 ± 2.91(7) (3.41–9.88)
4	26.45 ± 17.10(10) (4.86–65.53)	8.82 ± 8.91(8) (2.92–30.43)
6	17.18 ± 10.08(9) (3.92–38.01)	5.19 ± 3.26(10) (2.85–9.09)
12	8.27 ± 5.75(12) (2.72–18.87)	3.28 ± 1.64(11) (0.21–6.51)
24	8.01 ± 4.17(5) (2.28–15.68)	6.24 ± 4.38(4) (2.33–12.54)

## Discussion

Although vancomycin does not penetrate the BBB with high permeability, it is currently still a standard antibiotic protocol for treating intracranial infection caused by MRSA and MRCoNS after craniotomy. Norvancomycin has been considered to have a comparable antibacterial spectrum and anti-bacterial activity to those described for vancomycin. It had been widely used in preventing and treating gram-positive infection in China, although is not available therapeutically outside of China and pharmacokinetics-related studies conducted with this antimicrobial are rarely reported [11], especially the permeability of norvancomycin through the BBB in neurosurgery patients.

It has been previously found that damage to the BBB caused by neurosurgery tended to increase the vancomycin concentration in the CSF in the early stages after craniotomy, and intravenous infusion following a normal dose of 1 g for 1 h showed a peak CSF concentration of 6.24 ± 3.46 mg/L and a nadir of 2.55 ± 1.13 mg/L, which tended to achieve and exceed the 2 mg/L MIC_90_ of target bacterial MRSA [12].

In this study, we applied a normal daily dose of 1.6 g norvancomycin in a similar population in the early stages post-neurosurgery, and the results showed that in both the conventional and continuous administration groups, the CSF peak and trough concentrations were likely to exceed those of vancomycin. However, whether the BBB permeability of norvancomycin was higher than that of vancomycin warranted further investigation.

Referring to the literature [13–17], the MIC_90_ of norvancomycin to MRSA and MRCoNS were 0.5–2 and 1–4 mg/L, respectively, while that to *Staphylococcus epidermidis* could be as high as 4 mg/L. Thus, this study revealed that applying a normal dose of norvancomycin in the early stages post-neurosurgery tended to obtain an effective concentration to the above target bacteria in CSF by both conventional and continuous administration groups. However, the drug concentration in the CSF tended to present a decreasing trend with time, which might indicate that the permeability of the drug had declined with gradual repair of the BBB. Further studies are needed to investigate the temporal durability of the damage to the BBB and how long a higher concentration of norvancomycin will be retained in CSF.

Also, a significant advantage of continuous administration was not observed, which needs further investigation. In addition, this study adopted a normal dose (1.6 g/d) of norvancomycin, which showed that an effective concentration could be obtained in CSF in the early stages after craniotomy. Previous results showed that the recommended dose of vancomycin should be gradually increased in treating intracranial infection because of the “MIC shift” [18]. This is caused by gradual repair of the BBB and deteriorating drug resistance. However, whether it is necessary and safe to increase the dose of norvancomycin in treating intracranial infection requires further study.

In this study, a relative large standard deviation of drug concentration was obtained, which might be associated with the errors caused by deviation of sampling time, transportation, storage and detection of the specimens as well as a relatively small sample size. However, more importantly, it might have been due to a failure to administer a dose according to body weight since the degree of BBB permeability might differ in different patients. Therefore, studies with improved quality control and a larger sample size are expected.

In summary, this study preliminarily investigated the serum concentrations and CSF permeability with different administration methods of norvancomycin. These studies also provided preliminary clinical pharmacological evidence for assessing its value in preventing intracranial infection after neurosurgery.

## Conclusions

It indicated that the drug concentrations in the blood and CSF could obtain and exceed the MIC_90_ of the target bacteria, such as MRSA in both conventional and continuous administration groups, when applying a standard dose of norvancomycin in an early stage after neurosurgery. However, we failed to observe significant advantages of a continuous administration method over the conventional administration method. This study is a pilot research of norvancomycin used in neurosurgical individuals, which needs to be confirmed by further large-scale studies
